# Diurnal Cortisol Levels and Subjective Ratings of Effort and Fatigue in Adult Cochlear Implant Users: A Pilot Study

**DOI:** 10.1044/2019_AJA-19-0009

**Published:** 2019-08-20

**Authors:** Robert T. Dwyer, René H. Gifford, Fred H. Bess, Michael Dorman, Anthony Spahr, Benjamin W. Y. Hornsby

**Affiliations:** a Department of Hearing and Speech Sciences, Vanderbilt University Medical Center, Nashville, TN; b Department of Otolaryngology, Vanderbilt University Medical Center, Nashville, TN; c Department of Speech and Hearing Science, Arizona State University, Tempe; d Advanced Bionics, LLC, Valencia, CA

## Abstract

**Purpose:**

There is a growing body of literature that suggests a linkage between impaired auditory function, increased listening effort, and fatigue in children and adults with hearing loss. Research suggests this linkage may be associated with hearing loss–related variations in diurnal cortisol levels. Here, we examine variations in cortisol profiles between young adults with and without severe sensorineural hearing loss and examine associations between cortisol and subjective measures of listening effort and fatigue.

**Method:**

This study used a repeated-measures, matched-pair design. Two groups (*n* = 8 per group) of adults enrolled in audiology programs participated, 1 group of adults with hearing loss (AHL) and 1 matched control group without hearing loss. Salivary cortisol samples were collected at 7 time points over a 2-week period and used to quantify physiological stress. Subjective measures of listening effort, stress, and fatigue were also collected to investigate relationships between cortisol levels, perceived stress, and fatigue.

**Results:**

Subjective ratings revealed that AHL required significantly more effort and concentration on typical auditory tasks than the control group. Likewise, complaints of listening-related fatigue were more frequent and more of a problem in everyday life for AHL compared to the control group. There was a significant association between subjective ratings of listening effort and listening-related fatigue for our AHL, but not for the control group. In contrast, there was no significant difference in cortisol measures between groups, nor were there significant associations between cortisol and any subjective measure.

**Conclusions:**

Young AHL experience more effortful listening than their normal hearing peers. This increased effort is associated with increased reports of listening-related fatigue. However, diurnal cortisol profiles were not significantly different between groups nor were they associated with these perceived differences.

For some individuals with hearing loss, the simple act of listening can be mentally demanding. Those with hearing loss may be required to expend greater effort listening and devote more cognitive resources to listening than individuals without hearing loss (Pichora-Fuller et al., 2016). This is especially true in noisy environments where more top-down processing is required to fill in gaps in the conversation that they did not hear (e.g., Gosselin & Gagné, 2011; Picou, Ricketts, & Hornsby, 2013). There is a growing body of literature that suggests a linkage between impaired auditory function, increased listening effort, and fatigue in children and adults with hearing loss (e.g., Alhanbali, Dawes, Lloyd, & Munro, 2017; Bess & Hornsby, 2014; Hornsby, 2013; Hornsby et al., 2017; Hornsby & Kipp, 2016; Hornsby, Werfel, Camarata, & Bess, 2014; Kramer, Kapteyn, & Houtgast, 2006; Nachtegaal et al., 2009; Werfel & Hendricks, 2016). The underlying mechanisms responsible for these associations are unclear, but research suggests sustained stress, potentially resulting from the increased effort applied during listening, may play a role (Bess et al., 2016; Kramer, Teunissen, & Zekveld, 2016; Mackersie, MacPhee, & Heldt, 2015).

Cortisol, a hormone secreted by the adrenal gland, is an important biomarker of stress that has been used to study the body's response to stress-inducing events (Hellhammer, Wüst, & Kudielka, 2009). During a stressful event, complex interactions between the hypothalamus, the pituitary gland, and the adrenal cortex (i.e., the HPA axis) occur, resulting in a release of cortisol into the bloodstream. One function of cortisol is to modulate access to sugars in the blood stream and thus energy reserves. A rapid release in cortisol in response to a stressful event can provide an increase in energy critical for responding to the stressful event (i.e., the fight or flight response). Given normal HPA function, cortisol levels in the body follow a diurnal cycle. Cortisol levels increase sharply upon awakening, a pattern referred to as the *cortisol awakening response* (CAR). In typical adults and children, the CAR often results in a doubling of cortisol levels within the first 30 min after wakening, and as discussed below, variations in the CAR have been associated with a variety of psychological and physiological disorders. Following this peak, cortisol levels steadily decrease as the day wears on and rise again during sleep (Pruessner et al., 1997).

Activation of the HPA axis is necessary for everyday functioning; however, deviations from the normal pattern have been observed in healthy individuals (e.g., Stone et al., 2001) and in those with stress-related disorders (Jerjes, Cleare, Wessely, Wood, & Taylor, 2005; Pruessner, Hellhammer, & Kirschbaum, 1999). While individual variability is a hallmark of cortisol studies, common deviations include (a) variations in overall cortisol production throughout the day and (b) variations in the CAR. For example, a blunted or flattening of the diurnal cortisol pattern, combined with an increase in overall salivary cortisol production (hypercortisolism), as measured by the area under the cortisol profile (area under the curve [AUC]), has been observed in adults and children with severe depressive symptoms (Linkowski et al., 1985; Van den Bergh & Van Calster, 2009). In contrast, blunted cortisol profiles, along with a reduction in overall cortisol production (hypocortisolism), have been observed in individuals suffering from chronic fatigue syndrome (e.g., Jerjes et al., 2005).

In addition to an overall increase or reduction in cortisol levels, atypical CARs have also been observed among individuals experiencing high levels of stress and fatigue. For example, Pruessner et al. (1999) found that teachers who scored high on a measure of “burnout” had reduced cortisol levels upon awakening, compared to teachers with low burnout scores. However, results in the “burnout” literature are variable (see Sonnentag, 2006, for discussion), and the opposite effect (i.e., higher cortisol levels upon awakening) has also been observed (cf. De Vente, Olff, Van Amsterdam, Kamphuis, & Emmelkamp, 2003; Grossi et al., 2005). In addition, in some populations (e.g., burnout, hearing loss), the twofold increase in cortisol levels generally associated with the CAR is reduced or absent (Bess et al., 2016; De Vente et al., 2003; Grossi et al., 2005). The brief literature described above shows that the CAR and diurnal cortisol patterns can provide insight into the bodies' response to stress and fatigue.

Research suggests that, compared to peers without hearing loss, individuals with hearing loss may experience increased stress due, in part, to more frequent effortful listening (Alhanbali et al., 2017; Bess et al., 2016; Hasson, Theorell, Wallén, Leineweber, & Canlon, 2011; Kramer et al., 2006; Nachtegaal et al., 2009). However, studies examining the physiological response to that stress are limited. Recently, investigators have used cortisol to examine stress resulting from short-term, challenging, listening situations. For example, Rance, Chisari, Saunders, and Rault (2017) measured salivary cortisol levels in school-age children with autism spectrum disorder, some of which also had hearing loss. Cortisol measures were taken before and after a 20-min, structured listening sessions. During these sessions, children completed three challenging, listening-in-noise tasks. The listening tasks were completed twice, on separate days, once with and once without an Frequency Modulation (FM) system (ear-level and sound-field systems) to improve the signal-to-noise ratio. Results revealed an interaction between FM usage and time (pre- vs. posttesting) with a larger difference in cortisol levels between the FM and no-FM conditions posttesting. That is, when completing testing without the FM system activated, cortisol levels increased over the course of testing. However, when the children were tested using the FM system (personal FM or sound-field FM) levels decreased slightly, suggesting a reduction in listening-related stress with an improvement in signal-to-noise ratio provided by the FM system.

Kramer et al. (2016) also evaluated the effects of listening demands on cortisol levels and found no such effect of background noise or hearing loss in a sample of adults with (*n* = 10) and without (*n* = 10) hearing loss. Participants in the study of Kramer et al. completed two speech tests, one in quiet and one in noise. Salivary cortisol samples were obtained at four time points: once before testing started, two more times during speech testing, and once after each speech task. After completing both speech tasks, all participants completed a visual linguistic closure test. The fourth saliva sample was obtained after this visual task. In contrast to the study of Rance et al. (2017), cortisol levels were not sensitive to variations in task difficulty due to background noise. However, the small sample size and differences in experimental design make comparisons between studies difficult.

Hicks and Tharpe (2002) were the first to examine the effects of hearing loss on diurnal cortisol levels. They sampled cortisol levels, twice a day on two separate days, in a small group of school-age children (aged 5–11 years) with and without mild–moderate hearing loss (*n* = 10 per group). The first sample was collected in the morning (about 9:00 a.m.), and the second was collected near the end of the school day (about 2:00 p.m.). Average cortisol levels based on AUC measures were higher for the children with hearing loss compared to the control group, although the differences were not statistically significant. However, because only two samples per day were collected, this provided only a gross measure of cortisol production and did not allow examination of the CAR. In more recent work, Bess et al. (2016) used a more precise sampling procedure to better define the diurnal profile and the CAR in school-age children with mild-to-severe hearing loss (aged 6–12 years). They obtained saliva samples six times per day on two separate days. The collection times were chosen to allow measurement of the CAR (three samples within 60 min of awakening) and the remainder of the diurnal profile (samples obtained midmorning, afternoon, and evening). Results revealed that children with hearing loss (*n* = 32) had significantly elevated cortisol levels upon awakening and a shallower CAR compared to the control group (*n* = 28). The authors suggest the higher cortisol levels at awakening and a suppressed CAR support the hypothesis that children with hearing loss experience higher stress during the day and may be at an increased risk for stress-related disorders. This hypothesis is also supported by recent work showing that adults and children with hearing loss experience increased fatigue compared to control groups and/or normative data (Alhanbali et al., 2017; Hornsby & Kipp, 2016; Hornsby et al., 2017). Importantly, Bess et al. (2016) also found that mean cortisol levels increased with age, suggesting increased risk for stress and fatigue for children entering the upper elementary and middle school years.

Thus, the purpose of this pilot study was to examine the relationship between hearing loss, stress (measured via salivary cortisol levels), and fatigue. Our target populations were college-age adults with severe-to-profound hearing loss and a matched control group of adults without hearing loss. We focused on this group because (a) to date, there has been no work using salivary cortisol to assess listening-related stress and fatigue in college-age adults with hearing loss (AHL) and (b) college-age students may have an increased risk for listening-related stress and fatigue. For example, as mentioned above, Bess et al. (2016) showed cortisol levels in children increased with age. Likewise, Gordijn, Cremers, Kaspers, and Gemke (2011) found that subjective ratings of fatigue were greater among adolescents (13–18 years) compared to younger children (5–12 years).

Our primary research questions were as follows: (a) Compared to college-age adults with no hearing loss, does severe–profound hearing loss affect biological stress, as measured via cortisol levels? (b) Are there associations between cortisol levels and subjective ratings of stress, effort, and fatigue in this group? To answer these questions, salivary cortisol samples were collected throughout the day and used to evaluate overall cortisol production (i.e., via analysis of the AUC), cortisol levels upon awakening, and the CAR. In addition, subjective questionnaires were used to compare stress, effort, and fatigue between groups.

## Method

### Participants

All procedures were reviewed and approved in accordance with Vanderbilt University Institutional Review Board (IRB 130773). A total of 16 adults, with and without hearing loss, participated in this study (see Table 1). Eight participants (seven women, one man) had long-standing severe-to-profound sensorineural hearing losses (six cochlear implant [CI] recipients and two hearing aid [HA] users) and were enrolled in AuD programs around the country. AuD students were recruited via letters distributed by the Student Academy of Audiology. Almost 1,400 recruitment letters were distributed. Those with severe-to-profound hearing loss who were interested in participating and met our inclusion criteria (see below) were mailed a consent form, which they completed and returned prior to starting any study procedures. The mean age of participants with hearing loss was 24.3 years (range: 23–32 years). The following device configurations were represented: one unilateral CI, four bilateral CI, one bimodal (CI + HA), and two bilateral HA. The median age of initial intervention to address prelingual hearing loss was 1.5 years of age. All individuals with hearing loss used some sort of assistive technology. Three individuals used communication access real-time translation exclusively. All other participants used a combination of services including communication access real-time translation (*n* = 6), FM system (*n* = 4), note taker (*n* = 1), or preferential seating (*n* = 4).

**Table 1.** Demographic information of individuals with hearing loss (HL) and normal hearing (NH) controls.

	HL group (*n* = 8)							NH group (*n* = 8)		
Participant	Age (years)	Gender	Etiology	Device configuration	Age hearing loss identified (years)	Age at implantation first implant (years)	Age at implantation second implant (years)	Participant	Age (years)	Gender
HL1	32	Male	Meningitis	Bilateral CI	21	21	21	NH1	31	Male
HL2	23	Female	Connexin	Bilateral CI	1	1	20	NH2	23	Female
HL3	23	Female	Unknown	Bimodal	1	8	n/a	NH3	23	Female
HL4	23	Female	Usher	Bilateral CI	1	4	15	NH4	23	Female
HL5	23	Female	Unknown	Bilateral HA^a^	8	n/a	n/a	NH5	25	Female
HL6	23	Female	Pendred	Bilateral HA^a^	5	n/a	n/a	NH6	22	Female
HL7	23	Female	Unknown	Bilateral CI	2	3	17	NH7	23	Female
HL8	24	Female	Unknown	Bimodal	2	18	n/a	NH8	22	Female
*M*	24.25	7 Female	n/a	n/a	5.13	9.17	18.25		24	7 Female

*Note.* Each individual with HL was matched to an NH control within their cohort. In this table, HL1 is matched with NH1, HL2 with NH2, …, etc. CI = cochlear implant; HA = hearing aid; n/a = not applicable.

a Denotes a sensorineural HL bilateral and severe-to-profound in nature.

In addition, a matched-pair control group (seven women) was created by having each participant with hearing loss recruit a control participant (i.e., without hearing loss) from their own cohort of AuD students. The mean age of the control group was 24.0 years (range: 22–31 years). All control group participants had audiometric thresholds of < 20 dB HL at 500, 1000, 2000, and 4000 Hz.

Exclusionary criteria included (a) taking medications that affect the substances that stimulate the HPA axis (e.g., fluoxetine, paroxetine, sertraline); (b) taking medications that influence the subjective experience of stress, novelty, threat, or pain (e.g., hydrocodone, oxycodone, acetaminophen); (c) taking medications that may influence salivary composition (e.g., tizanidine, clonidine, lofexidine); (d) taking oral, nasal, topical, or ophthalmic corticosteroids (e.g., betamethasone, fluticasone, hydrocortisone); (e) having a learning disability; and (f) having a chronic medical condition that affects stress or fatigue (e.g., depression, anxiety).

### Salivary Cortisol Sample Collection Procedures

Seven samples were assayed from each participant. All samples were self-collected. All participants were provided directions on how to use the sample collection device from the manufacturer of the device (Esoterix). Samples were collected at “wake time” (T0), defined as the moment the participant opened their eyes and before their feet touch the ground, or when they opened their eyes and were ready to get up, or as soon as they were aware of being awake for the day and would not go back to sleep. Subsequent samples were taken 30–45 min after “wake time” (T1) and 12 hr after waking (T2) to allow for an AUC calculation. This protocol was repeated on the same day of the week, 1–2 weeks later. In addition, to capture potential differences in specific stress event–related fluctuations in cortisol levels, all participants provided a single saliva sample taken within 10 min after completing a graduate-level lecture that lasted at least 60 min. Because cortisol levels can vary in novel versus familiar situations (e.g., Frankenhaeuser & Lundberg, 1977; Hellhammer, Heib, Hubert, & Rolf, 1985), participants were instructed not to collect cortisol samples on days in which stress was considered atypical. We queried participants about their psychological state and the characteristics of their “most stressful” event on collection days using questions from the MacArthur salivary cortisol protocol (Stewart & Seeman, 2000). Specifically, on each day cortisol samples were collected, participants recorded their wake time and reported how excited and/or anxious they were upon awakening. They also rated their perceived stress and pressure on sample collection days.

### Subjective Measures of Stress, Listening Effort, and Fatigue

At the end of each saliva collection day, participants completed a series of online questionnaires assessing their stress, listening effort, and fatigue. Responses were entered and managed using REDCap electronic data capture tools hosted at Vanderbilt University (Harris et al., 2009). Subjective ratings of fatigue and vigor were assessed using subscales from the Profile of Mood States (POMS; McNair, Lorr, & Droppleman, 1971). The POMS provides a valid, reliable measure of general fatigue and vigor; however, it was not designed to assess issues of listening-related fatigue. Therefore, we developed a three-item questionnaire to specifically assess the impact of listening-related fatigue on their lives. Participants responded to the following statements using a 5-point (0–4) Likert scale: “Difficulty in listening causes me to become physically or emotionally tired” and “Fatigue and low energy due to difficulty in listening are major problems in my life.” Response options included “not at all,” “a little,” “moderately,” “quite a bit,” and “extremely.” Participants also responded to the question “How often do you feel physically or emotionally tired due to difficulty in listening?” using a 6-point (0–5) Likert scale. Response options included “once a month,” “once every 2 weeks,” “once a week,” “three to four times a week,” and “more than once a day.”

Finally, given the association between perceived listening effort and fatigue (Alhanbali et al., 2017), participants were also queried about their listening effort experienced in class and clinic. Specifically, items from Section 2 of the Amsterdam Checklist for Hearing and Work (Kramer et al., 2006) were modified to ask participants about the frequency (five items) and effort (five items) they applied to detect auditory signals and engage in conversation in class or clinic (rather than work) settings. Responses to frequency and effort items were coded using a 4-point (0–3) Likert scale, with 0 = *almost never* or *no effort* and 3 = *almost always* or *very much effort* for the frequency and effort items, respectively. Items were summed to create an overall “frequency of listening demands” and “effort in listening” scores. Scores ranged from a minimum of 0 (listening demands “almost never” occurred and required “no effort”) to a maximum score of 15 (listening demands were “almost always” present and required “very much effort”).

## Statistical Analyses

Cortisol data were first converted from μg/dL to the more traditional unit nmol/L. The distribution of cortisol levels is positively skewed; therefore, values were log-transformed (base 10) before analyses (Keene, 1995). An effect size *r* is reported for planned comparisons where an *r* of .10, .30, or .50 represent a small, medium, or large effect, respectively (Field, 2009). A preliminary analysis of variance (ANOVA) indicated that cortisol samples within groups were not different between days, *F*(1, 14) = 0.001, *p* = .97, *r* = .008. Therefore, we used data from each time point on both days in a repeated-measures fashion to provide more stable estimates of cortisol changes over time. A series of repeated-measures ANOVAs were used to examine differences in salivary cortisol levels upon awakening, the slope of the CAR, and total salivary cortisol production between groups (adults with and without hearing loss). Overall salivary cortisol output was estimated for each test day using AUC. An AUC for each participant was calculated using salivary cortisol levels obtained upon awakening (T0), 30–45 postawakening (T1), and 12 hr postawakening (T2). The AUC was calculated using Formula 1 from Pruessner, Kirschbaum, Meinlschmid, and Hellhammer (2003) and used to compare total salivary cortisol production between groups. Unless otherwise stated, Mann–Whitney tests were used to evaluate differences in subjective measures between groups. Correlation analyses (Pearson or Spearman rho as appropriate) were used to assess relationships between various physiological and subjective measures.

## Results

### Salivary Cortisol Levels: A Biomarker of Stress

**Figure 1.**
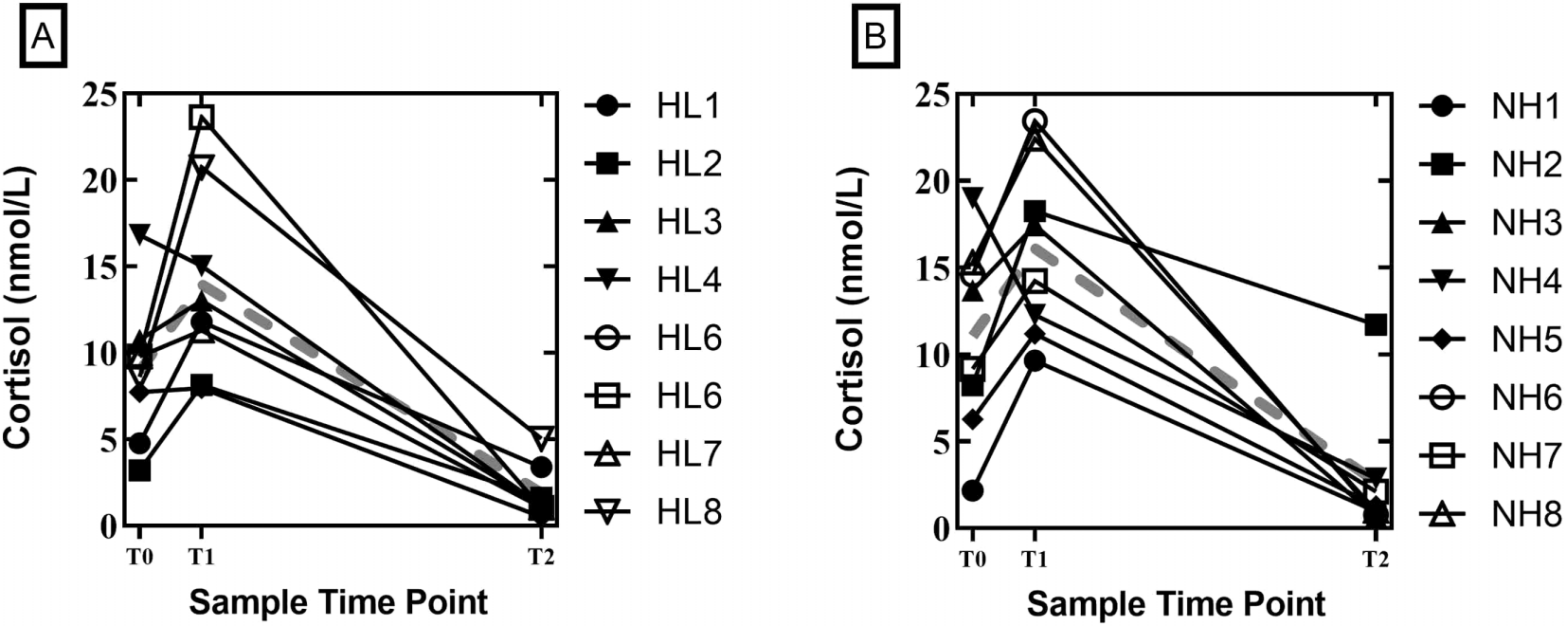
Individual salivary cortisol levels (in nmol/L) as a function of sample time (T0, T1, T2) for (A) individuals with hearing loss (HL) and (B) normal hearing controls (NH). The gray, dashed line represents the group mean. Samples were collected at “wake time” (T0), defined as the moment the participant opened their eyes and before their feet touched the ground, or when they opened their eyes and were ready to get up, or as soon as they were aware of being awake for the day and will not go back to sleep. Subsequent samples were taken 30–45 min after “wake time” (T1) and 12 hr after waking (T2) to allow for an area under the curve calculation. A preliminary analysis of variance indicated that cortisol samples within groups were not different between days. Therefore, we used data from each time point on both days in a repeated-measures fashion to provide more stable estimates of cortisol changes over time.

Of primary interest in this study was whether diurnal cortisol patterns, a physiological marker of stress, varied between college-age adults with and without hearing loss. Mean cortisol levels, averaged across days, at each cortisol collection time point are shown in Figure 1. As noted earlier, Bess et al. (2016) found elevated salivary cortisol levels upon awakening in school-age children with hearing loss compared to a control group of children without hearing loss. Importantly, this between-groups difference in awakening cortisol levels increased with age. To see if this difference was maintained in college-age AHL, we compared the Day 1 and Day 2 salivary cortisol levels obtained upon awakening from our college-age AHL (8.9 nmol/L) to our control group (12.2 nmol/L). Results of a mixed-model, repeated-measures ANOVA revealed no main effect of day and no significant difference in awakening (T0) salivary cortisol levels between groups, *F*(1, 14) = 0.13, *p* = .73, *r* = .095 (see Figure 1).

Potential differences in CARs between groups were also examined using a mixed-model repeated-measures ANOVA. Independent variables were sample day (Day 1 or 2), sample time point (T0 and T1), and group (CI users and adults without hearing loss). The CAR is evidenced in the change in cortisol levels from T0 (awakening level) to T1 (30–45 min postawakening). Prior work by Bess et al. (2016) also revealed a shallower CAR in children with hearing loss. Thus, of interest in this analysis was a potential interaction between group and sample time. Results revealed a significant main effect of time, *F*(1, 14) = 15.1, *p* = .002, *r* = .72, with mean cortisol levels, averaged across groups, increasing postawakening by approximately 50%. Specifically, mean cortisol levels, averaged across groups, increased from approximately 10.0 to 15.0 nmol/L when measured at awakening (T0) and again at 30–45 min postawakening (T1), respectively. However, there was no main effect of group, *F*(1, 14) = 0.35, *p* = .57, *r* = .15, and in contrast to that of Bess et al. (2016), the Group × Time interaction was not significant, *F*(1, 14) = 0.012, *p* = .91 (see Figure 1).

In addition, we completed a mixed-model ANOVA on AUC data to compare overall cortisol production between groups. Results revealed no significant difference in overall salivary cortisol production between our hearing loss group (1.98 nmol/L) and the control group (2.05 nmol/L), *F*(1, 14) = 0.77, *p* = .40, *r* = .23.

Finally, to explore between group differences in response to a potential stress-inducing event, we compared cortisol levels obtained immediately after participants with hearing loss and their matched pair completed a 60-min graduate-level course. A paired-samples *t* test revealed no significant differences, *t*(7) = 0.28, *p* = .79, *r* = .10, in cortisol levels of AHL (*M* = 0.46 nmol/L) and their matched pairs (*M* = 0.42 nmol/L).

### Subjective Assessments

#### MacArthur Salivary Cortisol Protocol Responses

Responses to MacArthur Salivary Cortisol Protocol questions were used to assess differences in participant psychological state and characteristics of their primary stress event on saliva sample collection days. A series of mixed-model, repeated-measures ANOVAs were used to make between-groups (hearing loss vs. no hearing loss) comparisons of participants wake times and the time of day and duration of their primary stressful event. Results revealed no main effect of Group or Significant Group × Day interactions (all *p*s > .05) for any of these MacArthur measures. To compare participants' perceptions of happiness, anxiety, stress upon awakening, and the stress of a primary event, we first compared ratings across saliva collection days. Results of a series of Wilcoxon signed-ranks test revealed no significant difference across days (all *p*s > .05), so ratings were summed to create a composite score. A series of Mann–Whitney tests using composite scores indicated that our participants with hearing loss felt no more anxious (*U* = 15.5, *z* = −1.9, *p* = .054, *r* = −.48), excited (*U* = 30.5, *z* = −0.17, *p* = .87, *r* = −.04), busy (*U* = 27.5, *z* = −0.49, *p* = .62, *r* = −.12), or stressed (*U* = 27.0, *z* = −0.55, *p* = .58, *r* = −.14) compared to our control group.

#### Assessing Listening Effort in Class and Clinic

**Figure 2.**
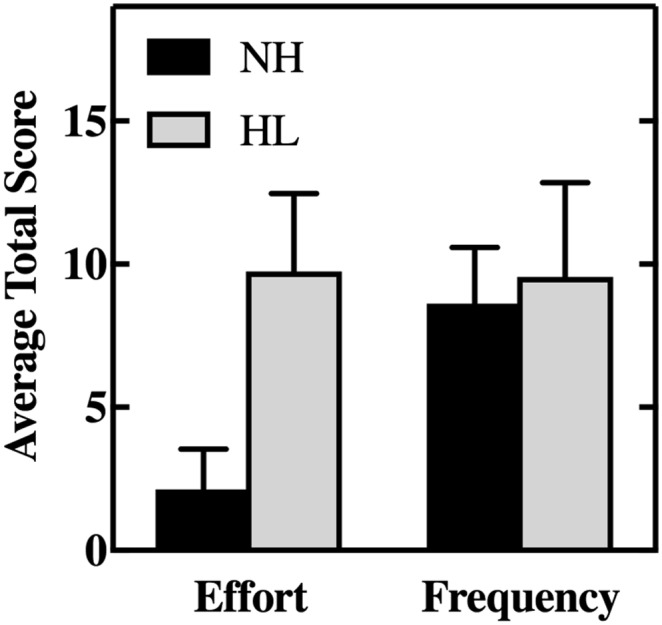
Average total effort and frequency as measured by the Amsterdam Checklist for Hearing and Work. Participants reported the effort and frequency necessary to detect auditory signals and engage in conversation in class or clinic settings. Participant reported frequency and effort of detecting sounds, following conversation in noise, following conversation in quiet, distinguishing between sounds, and localizing sounds in a class or work environment. Responses to were coded using a 4-point (0–3) Likert scale, with 0 = *almost never* and 3 = *almost always*, and responses were summed to give a total score. A greater total score indicates more effort and greater frequency. Error bars represent 1 *SD*. NH = normal hearing; HL = hearing loss.

We used responses from a modified version of the Amsterdam Checklist for Hearing and Work to compare “frequency of listening demands” and “effort in listening” between AHL and their matched pairs. We first used a Wilcoxon signed-ranks test to compare summed scores obtained on the first and second sample days. There was no difference in the frequency of listening demands between days (*z* = −0.28, *p* = .78, *r* = .07). However, effort ratings on the first sample day (*Mdn* = 6.5) were significantly higher (*z* = −2.31, *p* = .21, *r* = .58) than those obtained on the second sample day (*Mdn* = 4.0). Therefore, we analyzed between-groups effort ratings and for consistency frequency ratings, separately for each sample day. A series of Mann–Whitney *U* tests revealed no significant difference in frequency of listening demands between groups (Day 1: *z* = −0.16, *p* = .87, *r* = .04; Day 2: *z* = −0.58, *p* = .56, *r* = .15). However, ratings of the amount of effort required on listening tasks were significantly higher on both days (Day 1: *z* = −3.38, *p* = .001, *r* = .85; Day 2: *z* = −3.33, *p* = .001, *r* = .83) for the hearing loss group (median ratings of 10 and 8.5 on Days 1 and 2, respectively) compared to their age-matched controls without hearing loss (median ratings of 2 and 1.5 on Days 1 and 2, respectively). Figure 2 shows the mean summed scores for total effort (left) and frequency (right) of listening demands averaged across sample days for our CI users and their age-matched controls.

#### Assessing Subjective Fatigue and Vigor

**Figure 3.**
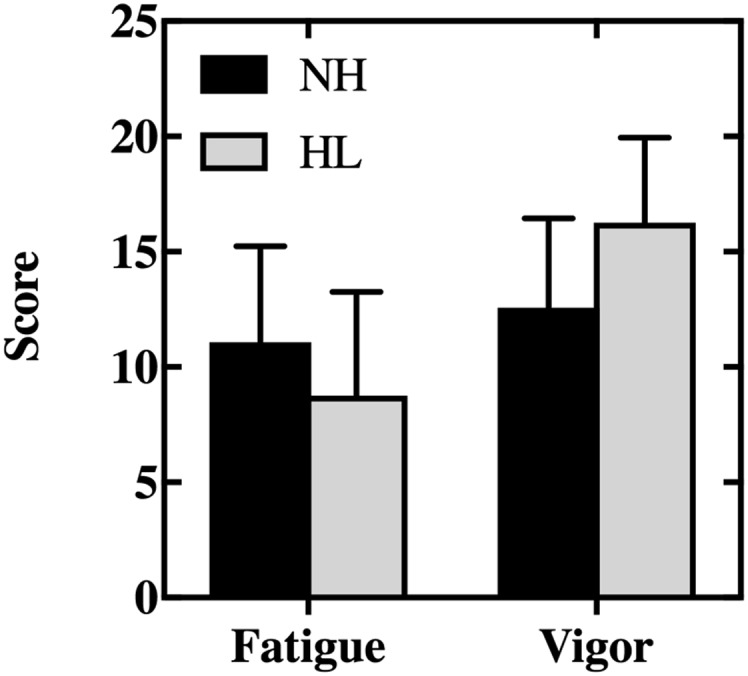
Mean ratings of fatigue and vigor reported by individuals with hearing loss (HL) and those with normal hearing (NH) as measured by POMS. “Fatigue” or “vigor” represent the sum (total score) of a series of questions that probe into how a person felt over the last week, including the date the questionnaire was given. Higher ratings indicate greater feelings of fatigue or vigor. Error bars represent 1 *SD*.

We used the POMS to assess participants' long-term general fatigue and vigor. First, we used a Wilcoxon signed-ranks test to compare fatigue and vigor ratings between sample collection days. No between-days differences in fatigue (*z* = −0.44, *p* = .66, *r* = .11) or vigor (*z* = −1.32, *p* = .19, *r* = .33) were observed; therefore, ratings were collapsed across days for subsequent analyses. Next, we used a series of Mann–Whitney *U* tests to compare ratings of fatigue and vigor between our hearing loss and control participants (see Figure 3). In contrast to expectations, our control group (college-age adults without hearing loss) reported less vigor (median rating =12.5) and more fatigue (median rating =10.5) than our hearing loss group (median ratings of 15.5 and 7.0 for vigor and fatigue, respectively). However, these differences were not statistically significant (fatigue: *U* = 20.5, *z* = −1.21, *p* = .23, *r* = .30; vigor: *U* = 16.5, *z* = −1.64, *p* = .10, *r* = .41). It is worth noting that the fatigue and vigor ratings of both groups in the current study are quite similar to existing normative data for college-age adults (*M* = 10.6 and 15.6 for fatigue and vigor, respectively; McNair & Heuchert, 2010). Finally, Hornsby and Kipp (2016) found AHL had an increased risk for experiencing severe fatigue and vigor deficits. Therefore, we examined the prevalence of severe problems in our sample. Severe fatigue and vigor deficits are defined as scores exceeding normative means by ± 1.5 *SD*s (McNair & Heuchert, 2010). For college-age adults, as sampled in the current study, severe deficits correspond to scores of > 20 and < 6 for fatigue and vigor, respectively. However, in contrast to the study of Hornsby and Kipp, no respondents from either group reported severe fatigue on either sample day. Only one participant, one without hearing loss, reported a severe vigor deficit (vigor score = 4), and that occurred on only one of the two sample days. Thus, severe fatigue and vigor deficits, as measured using the POMS, were not a problem for these participants.

#### Listening-Related Fatigue

**Figure 4.**
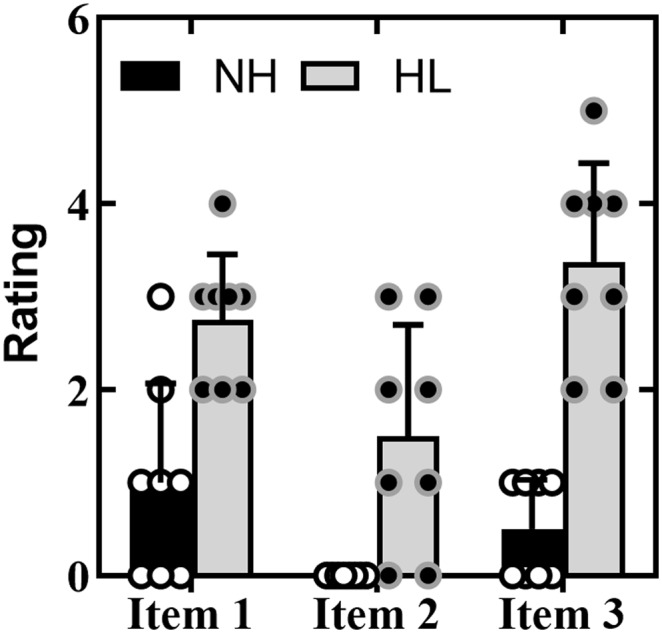
The range of responses to our listening-related fatigue questions, as a function of group. Filled circles represented individual data for the normal hearing participants (NH; black) and the individuals with hearing loss (HL; gray). The columns represent the average rating. Error bars represent 1 *SD*. A higher score indicates an individual is physically and emotionally tired to a greater extent due to difficulty in listening (Item 1), that fatigue and low energy due to difficulty in listening were more of a major problem in their lives (Item 2), and that an individual was more often physically and emotionally tired due to difficulty in listening (Item 3).

A series of Mann–Whitney *U* tests were used to assess the impact of hearing loss on listening-related fatigue. Interestingly, results of these analyses contrasted with our POMS findings, which assessed general feelings of fatigue and vigor. When specifically queried, college-age adults with severe hearing loss reported that listening-related fatigue was much more of a problem for them compared to their age-matched controls without hearing loss. Specifically, participants with hearing loss reported (*Mdn* = 3; “quite a bit”) they were physically and emotionally tired to a greater extent due to difficulty in listening (*U* = 6.5, *z* = −2.76, *p* = .006, *r* = .69) than their matched pairs (*Mdn* = 1; “a little”). Also, compared to their matched controls, CI users reported that fatigue and low energy due to difficulty in listening were more of a major problem in their lives (*U* = 8.0, *z* = −2.91, *p* = .004, *r* = .73). It is worth noting that all eight participants without hearing loss responded “not at all” (0) to this question. In contrast, responses from adults with severe hearing loss varied from “not at all” (0) to “quite a bit” (3) with a median response rating of 1.5 (between “a little” and “moderately”). Finally, AHL also reported they were more often physically and emotionally tired due to difficulty in listening (*Mdn* = 3.5; between “once a week” and “three to four times a week”) compared to their matched pairs (*Mdn* = 0.5; between “not at all” and “once a month or less”; *U* = 0.00, *z* = −3.43, *p* = .001, *r* = .86). The range of responses to our listening-related fatigue questions, as a function of group, is shown in Figure 4.

### Associations Between Physiological Stress (Cortisol Levels) and Subjective Ratings of Effort and Fatigue

We used a series of Spearman rank correlations to examine associations between our various physiological measures of stress (awakening cortisol level, CAR, and AUC) and subjective ratings of effort (Amsterdam effort questions) and general fatigue and vigor (POMS ratings). We quantified the CAR as the difference in cortisol levels obtained 30–45 min postawakening and levels obtained upon awakening (i.e., T1–T0 cortisol levels). Association between cortisol measures and listening-related fatigue were explored using responses from our three listening fatigue questions. Listening effort ratings from sample collection Day 1 and Day 2 were analyzed separately given the significant difference in perceived effort between days (see the Listening-Related Fatigue section). Given the pilot nature of our study, we chose to explore these relevant associations without controlling for potential inflation of Type 1 errors. Despite this liberal criterion, results of these exploratory analyses revealed no significant correlation between any cortisol measure and any subjective measure of effort or fatigue (all *p*s > .05).

Prior research suggests an association between perceived listening effort in daily settings and ratings of long-term general fatigue (Alhanbali et al., 2017). Therefore, we also explored associations between listening effort (summed effort scores from the Amsterdam Checklist) and subjective ratings of listening-related fatigue based on our three questions. Given the large and significant differences in listening demands and listening-related fatigue seen between our hearing loss participants and their matched controls, associations were examined separately for each group. Again, due to the pilot nature of our study, no corrections were applied to control for potential inflation of Type 1 errors. Results revealed a positive correlation between everyday listening demands and listening-related fatigue for our adults with severe hearing loss (four of six comparisons were significant at *p* < .05) but not for their matched controls without hearing loss (all *p*s > .05). Specifically, listening demands were positively correlated with the degree to which difficulty in listening causes an individual to become physically or emotionally tired (Day 1: *r_S_* = .79, *p* = .02 and Day 2: *r_S_* = .74, *p* = .038 ); with how much a problem fatigue and low energy due to difficulty in listening is in an individual's life (*r_S_* = .77, *p* = .025 and Day 2: *r_S_* = .64, *p* = .089) and with how often a person felt physically or emotionally tired due to difficulty in listening (*r_S_* = .71, *p* = .051 and Day 2: *r_S_* = .80, *p* = .017).

## Discussion

Listening-related fatigue in AHL is poorly understood. In the primary authors' day-to-day interactions with AHL, a common report is how physically and mentally drained individuals with hearing loss are as a result of day-to-day interactions. This is of importance to the field of audiology if increased listening effort, stress, and subsequent fatigue lead to frequent activation of the HPA axis. Chronic activation has been implicated in the development of significant health conditions, including metabolic syndrome, cardiovascular disease, cognitive decline, and a variety of mood disorders and functional illnesses. The purpose of this research was to examine whether adults with severe hearing loss experience more physiological stress, as quantified by various cortisol measures (i.e., cortisol upon awakening, CAR, AUC) and greater fatigue than adults without hearing loss. A secondary purpose was to examine potential associations between various cortisol measures and subjective measures of listening effort and fatigue.

### Salivary Cortisol Levels

In contrast to our predictions, average awakening cortisol levels in our AHL group were slightly lower than cortisol levels for the normal hearing (NH) group, although the difference was not statistically significant. Likewise, no statistical differences in the CAR or AUC between groups were observed. Our findings are in line with the findings of Hicks and Tharpe (2002), who also did not observe an effect of hearing loss on diurnal salivary cortisol levels. They are, however, inconsistent with findings from Bess et al. (2016), who observed higher cortisol levels at awakening, a shallower CAR, and increasing cortisol levels with age among their children with hearing loss. These results suggest that children with hearing loss may experience higher stress during the day, which could put children with hearing loss at a higher risk for stress induced fatigue.

The lack of significant differences in cortisol measures between our groups of college-age participants may suggest that salivary cortisol, a physiological measure of stress thought to be associated with fatigue, is not sensitive enough to detect hearing loss–related stress in successful CI users, should they exist, in this age group. However, given the pilot nature of this study, it is important to acknowledge limitations that could have also affected our outcomes. For example, our cortisol sampling intervals (awakening, 30–45 min postwakening, and 12 hr postwakening) were quite sparse and thus not optimal for assessing the CAR. It is possible that collecting more samples during the day would increase our ability to precisely measure differences in cortisol levels between groups, should they exist. Bess et al. (2016) collected samples at six time points throughout the day, on two separate days, thus providing improved resolution compared to this study and that of Hicks and Tharpe (2002). In addition, it is important to note the relatively small sample size of the current study (*n* = 10 participants/group) could limit our ability to detect significant differences between groups. Given our small sample size, effect sizes of 1.49 or larger would be needed to achieve adequate statistical power (80%). According to Cohen (1992), small, medium, and large effect sizes are *d* = 0.2, 0.5, and 0.8, respectively, so our sample would be sensitive to only extremely large group differences.

In this study, salivary cortisol samples were also taken after a lecture lasting at least 60 min. We examined salivary cortisol levels from this time point and found no differences between groups. This finding was in agreement with that of Kramer et al. (2016), who found no effect of listening task difficulty on transient cortisol levels in a group of adults with and without hearing loss. However, they are inconsistent with the findings of Rance et al. (2017), who found that, when completing a challenging listening/comprehension session, children with autism (some with hearing loss and/or functional hearing deficits) were more likely to experience an increase in cortisol production when the listening conditions were more difficult (i.e., no FM system used) compared to an easier listening condition (i.e., when an FM system was used to improve understanding). Again, reasons for differences between studies are unclear; however, differences in study design may have played a role. For example, all participants in the study of Rance et al. completed the same listening/comprehension task and provided cortisol samples immediately before and after the task. In contrast, although all participants in the current study listened to a graduate-level lecture that lasted at least 60 min, not all participants listened to the same lecture. In addition, participants in the current study did not provide a baseline cortisol measure prior to the start of class. Thus, we cannot compare changes in cortisol levels following the listening task between our hearing loss and control groups.

In addition to some study design limitations inherent in a pilot study, it is also possible that a population bias is affecting our results. Specifically, study participants had extensive experience with their HAs and/or CIs and obtained significant benefit from them. The CI participants had, on average, 15.5 years of experience with their implant and were implanted at a young age. Additionally, three participants were deafened postlingually—potentially influencing their success with the implant. The stress and listening difficulties, as well as the required effort and subsequent fatigue of these situations, may be different for individuals with acute, acquired, or even progressive hearing losses. Future research should consider the duration, the etiology, and the impact of cochlear implantation on salivary cortisol levels in a larger sample.

In addition to being successful users of their technology, these participants chose to put themselves in a quite challenging listening situation (i.e., a graduate program in audiology). It is intuitive to think that if these difficult listening situations were very stressful and fatiguing that these individuals would have ultimately chosen different career path. Selecting a group more representative of the general population might also yield different results.

Finally, it is worth noting that, should these results be replicated in a much larger sample, the lack of a significant difference is an extremely positive finding. Such a result suggests that the increased listening effort commonly experienced by people with hearing loss may not universally contribute to increased physiological stress and its concomitant negative effects.

### Subjective Reports

A secondary aim of this pilot study was to examine subjective ratings of effort and fatigue in our sample and to investigate associations between cortisol levels, our physiological measure of stress, and these subjective measures. We did this using a generic measure of fatigue and vigor (POMS) and subjective scales focused on listening effort and listening-related fatigue (i.e., Amsterdam Checklist for Hearing and Work and study-specific listening effort/fatigue questions). In the current study, although AHL reported expending more “listening effort” than our control group, generic ratings of fatigue and vigor, as measured by the POMS, were not significantly different between groups. The lack of a significant difference in mean fatigue ratings between groups is consistent with some prior work using the POMS with older adults (Hornsby & Kipp, 2016). In that study, POMS fatigue and vigor ratings were collected from 149 adults with varying degrees of hearing loss and compared to normative data. In that study, consistent with the current study, the mean fatigue ratings of AHL were not significantly different than normative data. However, in contrast with the current study, significant differences in vigor ratings between the AHL and normative data were observed. In addition to examining differences in group means, Hornsby and Kipp also compared the prevalence of “severe” fatigue and vigor deficits, defined as ratings of > 1.5 *SD*s from mean values, between groups. Importantly, they found AHL were more than twice as likely to experience severe fatigue and more than 4.5 times as likely to report severe vigor deficits. Interestingly, no AHL in the current study reported severe fatigue or vigor problems.

The reasons for these inconsistent findings and the lack of group differences in POMS fatigue and vigor ratings in the current study are unclear. As mentioned above, differences in the target population, college-age adults who are successful in a highly competitive academic environment versus the general population, could play a role. It is also possible that the younger individuals in the current study had more experience adapting to their hearing loss and that their subjective perception of the impact of that hearing loss might be different than older adults or even younger children who have not yet learned to navigate their environment as an individual with a sensory deficit. However, the inconsistent findings may also reflect a weakness of generic fatigue measures for examining listening-related fatigue. This is supported by our finding that, when asked questions specifically targeting listening-related effort and fatigue (i.e., Amsterdam Checklist for Hearing and Work and study-specific listening effort/fatigue questions), AHL reported they experienced significantly more listening effort and listening-related fatigue than their NH age-matched controls.

In addition, using these listening-specific measures, we observed a significant correlation between subjective listening effort and listening-related fatigue. This finding is consistent with that of Alhanbali et al. (2017), who, also using listening-related items, found that listening effort and fatigue (measured using a generic fatigue scale) were increased in AHL compared to an NH control group. They also observed a significant correlation between the two constructs. Together, these findings suggest a weakness in the POMS for identifying problems of listening-related fatigue. This may reflect the nature of the POMS, which was designed to measure a variety of moods (including general fatigue and vigor) rather than specifically targeting listening-related issues. For example, the POMS uses general descriptors to assess fatigue (e.g., worn out, listless, weary, bushed) and vigor (e.g., lively, active, alert). This approach is useful for assessing general fatigue and vigor issues arising from a variety of factors (e.g., cancer, physical exertion). However, our results suggest these general descriptors may not be as sensitive to listening-related fatigue as items, such as used in this study, that target that issue. Regardless, inconsistent findings obtained using the POMS highlight the potential benefits of a questionnaire specifically designed to measure listening-related fatigue associated with hearing loss.

### Future Directions

Additional measurable benefits from cochlear implantation, such as reduced listening effort and listening-related fatigue, should be considered when assessing successful outcomes in other areas of the recipient's everyday life. The audiologist's largest measure of CI success has been and continues to be speech-based measures. However, we often see that clinical speech measures do not translate well to real-world benefit, highlighting the need for additional relevant outcome measures.

To be successful in an academically challenging, doctoral program, as were these study participants, requires an ability to cope with stress and adversity. As discussed above, the participants in this study were successful CI/HA users who had long-standing, successful experiences with their listening devices. It is possible that, by selecting this group as study participants, we have underestimated the potential impact of hearing loss on stress, effort, and listening-related fatigue.

Finally, it would also be interesting to follow these individuals over time to see how their perceptions of vigor and fatigue change as a result of electrical stimulation. These changes could be a useful health-related quality of life outcome measure, which is currently not included in the battery of tests clinicians use to assess outcomes.

## Conclusions

This is the first study to examine the relationship between diurnal salivary cortisol levels, subjective ratings of listening effort, and fatigue in college-age adults with severe-to-profound hearing loss. AHL experienced more effortful listening during their day and, as a result, reported more listening-related fatigue than their age-matched controls without hearing loss. Consistent with the literature (e.g., Hornsby, 2013; Mackersie et al., 2015), we observed a general lack of correlation between our objective and subjective measures of listening effort. This could reflect a limited sensitivity of salivary cortisol to listening-related effort and fatigue and/or that these measures simply reflect different components of listening effort and fatigue.

Currently, listening effort and fatigue can be evaluated clinically using questionnaires. These questionnaires can be used to quantify the amount of effort or difficulty in listening but ultimately reveal one's perception of the ease or effort involved (Gosselin & Gagné, 2011). Because of the nonauditory effects of hearing loss, a questionnaire specifically designed to measure listening-related fatigue in individuals with hearing loss would be beneficial.
